# Effect of predicted low suspend pump treatment on improving glycaemic control and quality of sleep in children with type 1 diabetes and their caregivers: the QUEST randomized crossover study

**DOI:** 10.1186/s13063-018-3034-4

**Published:** 2018-12-04

**Authors:** Ulrike Schierloh, Gloria A. Aguayo, Muriel Fichelle, Cindy De Melo Dias, Aljosa Celebic, Michel Vaillant, Katharine Barnard, Ohad Cohen, Carine de Beaufort

**Affiliations:** 1 Department of Pediatric Diabetes and Endocrinology, Clinique Pédiatrique, Centre Hospitalier, Luxembourg City, Luxembourg; 2 Luxembourg Institute of Health, Luxembourg City, Luxembourg; 3 Bournemouth University, Bournemouth, UK; 4 Medtronic Diabetes, Tolochenaz, Switzerland

**Keywords:** Type 1 diabetes, Sleep, Quality of life, Children, Caregiver, Sensor-augmented insulin pump, Flash glucose measurement

## Abstract

**Background:**

In attempting to achieve optimal metabolic control, the day-to-day management is challenging for a child with type 1 diabetes (T1D) and his family and can have a major negative impact on their quality of life. Augmenting an insulin pump with glucose sensor information leads to improved outcomes: decreased haemoglobin A1c levels, increased time in glucose target and less hypoglycaemia. Fear of nocturnal hypoglycaemia remains pervasive amongst parents, leading to chronic sleep interruption and lack of sleep for the parents and their children.

The QUEST study, an open-label, single-centre randomized crossover study, aims to evaluate the impact on time in target, in hypoglycaemia and hyperglycaemia and the effect on sleep and quality of life in children with T1D, comparing a sensor-augmented pump (SAP) with predictive low glucose suspend and alerts to the use of the same insulin pump with a flash glucose measurement (FGM) device not interacting with the pump.

**Methods/design:**

Subjects meeting the inclusion criteria are randomized to treatment with the SAP or treatment with an insulin pump and independent FGM for 5 weeks. Following a 3-week washout period, the subjects cross over to the other study arm for 5 weeks. During the week before and in the last week of treatment, the subjects and one of their caregivers wear a sleep monitor in order to obtain sleep data. The primary endpoint is the between-arm difference in percentage of time in glucose target during the final 6 days of each treatment arm, measured by a blinded continuous glucose measurement (CGM).

Additional endpoints include comparison of quantity and quality of sleep as well as quality of life perception of the subjects and one of their caregivers in the two different treatment arms.

Recruitment started in February 2017. A total of 36 patients are planned to be randomized. The study recruitment was completed in April 2018.

**Discussion:**

With this study we will provide more information on whether insulin pump treatment combined with more technology (SmartGuard® feature and alerts) leads to better metabolic control. The inclusion of indicators on quality of sleep with less sleep interruption, less lack of sleep and perception of quality of life in both children and their primary caregivers is essential for this study and might help to guide us to further treatment improvement.

**Trial registration:**

ClinicalTrials.gov, NCT03103867. Registered on 6 April 2017.

**Electronic supplementary material:**

The online version of this article (10.1186/s13063-018-3034-4) contains supplementary material, which is available to authorized users.

## Type 1 diabetes Background

Patients with type 1 diabetes (T1D) need lifelong insulin treatment and optimal metabolic control, which is essential to prevent short- and long-term complications [1]. To achieve optimal metabolic control, the day-to-day management is challenging for the children and their families and can have a major negative impact on their quality of life [2, 3].

Augmenting an insulin pump with glucose sensor information leads to improved outcomes. Whilst continuous interstitial glucose monitoring is associated with decreased haemoglobin A1c (HbA1c) levels and reduced time spent in hypoglycaemia in individuals with T1D using insulin pump therapy, better outcomes are associated with longer and continued use of the sensor [4]. Alerts are programmed and used in sensor-augmented pumps (SAPs) in order to inform patients and their caregivers about hypoglycaemic and hyperglycaemic events so that they can react quickly to such glycaemic excursions. However, alerts may be perceived as disturbing and may lead to diabetes distress and alert fatigue as well as continuous nocturnal awakenings [5].

Fear of nocturnal hypoglycaemia is common amongst parents of children with T1D, and it is associated with heightened vigilance by parents to regularly control their children’s blood sugar values or to check the sensor information during the night [6, 7]. This leads to chronic sleep interruption and to lack of sleep for the parents as well as for their children with diabetes [8]. Recent data show that 99% of parents of children with T1D perform blood glucose checks on their child during the night to ensure their safety whilst sleeping [9]. This highly prevalent chronic sleep interruption affects both adults with T1D and parents/carers of children with T1D, with resulting negative effects on their daily functioning and well-being [10]. Anxiety and fear of hypoglycaemia may have an impact on diabetes management and may complicate meeting glucose targets in patients with T1D [11, 12]. A recently published multicentre evaluation shows that SmartGuard® technology significantly reduced the risk for hypoglycaemia in paediatric diabetes patients without increasing HbA1c [13].

The MiniMed 640G® pump combines alerts with an automated insulin suspension; the pump suspends insulin infusion when the sensor glucose (SG) is within 3.9 mmol/l (70 mg/dl) above the low limit and predicted to be 1.1 mmol/l (20 mg/dl) or lower above the low limit in 30 min. Suspension lasts for a minimum of 30 min and until a 2-h suspension time is reached or the patient manually resumes basal rate infusion or auto-resumption occurs when the trend in glucose shifts and the SG is predicted to be 2.2 mmol/l (40 mg/dl) above the threshold in 30 min. Alerts can be set on or off; the low threshold alert is mandatory (SmartGuard®).

FreeStyle Libre**®** is another device that continuously measures the interstitial glucose levels. The results can only be obtained when the patient/caregiver actively scans the sensor (flash glucose measurement, FGM). No alerts are given when glucose values increase or decrease, nor will information be available when the sensor is not scanned, and data are lost when more than 8 h elapse between scans. No communication exists between this glucose measurement and the insulin pump. The advantage of the FGM is intermittent access to 24-h glucose profiles without disturbing alerts.

Without any alerts, however, the symptoms of high or low glucose levels may be missed, and intervention delayed.

The impact of these technologies on metabolic control has been studied before [14]. We are not aware of any study evaluating their impact on quality of sleep in children and their caregivers, using questionnaire and Actigraph® data.

## Objective

The objective of this study is to evaluate whether the SAP (MiniMed 640G®) with SmartGuard® feature increases time in glucose target and improves sleep quality and quantity and quality of life perception in patients with T1D and their primary caregivers, when compared with pump treatment with only continuous monitoring, FreeStyle Libre®.

## Methods

### Study design

In this open-label, single-centre, randomized two-period crossover study, based in the Children’s Hospital in Luxembourg, subjects with type 1 diabetes (6–14 years old, diabetes duration more than 6 months, on insulin pump for at least 6 months, HbA1c ≤ 11%) are randomized to treatment with SAP with the SmartGuard® feature (MiniMed® 640G) or treatment with insulin pump and independent interstitial glucose measurement (FreeStyle Libre®) for 5 weeks. Following a 3-week washout period, the subjects cross over to the other study arm for 5 weeks.

The subjects and one of their caregivers will wear a sleep monitor (Actigraph®) and complete a sleep diary the week before and during the last week of each treatment. This allows a comparison of the quality of sleep of the participants as well as the parents between the two treatment arms.

Randomization is performed by blocks of 8 with sequences A – B and B – A for the first and second periods respectively. Allocation is based on envelopes where the sequence code is concealed in advance. The order of the envelopes is determined by the randomization order and will allow allocation of the patient to one of the two sequences.

Reporting standards will be ensured by a rigorous quality process. The statistical process will include a statistical analysis plan approved by all members of the project team which will be applied for the statistical analysis of the study. Programming of the statistical analysis will be achieved and validated with a preliminary set of data and validated again when all data are available. Reporting will be done by the project statistician in a statistical report and validated by a second statistician.

The *primary endpoint* is the between-arm difference in percentage of time in glucose target during the final 6 days of each treatment arm, measured by a blinded continuous glucose measurement (CGM) (IPro2®).

*Secondary endpoints* include sleep duration, number of awakenings during the night and information about state of fatigue and activities in the daytime.

### Baseline and repeated assessment measures

Demographic variables are sex, age, income, education and socio-economic status; anthropometric variables are weight (kg) and height (m). Quality of life perception will be assessed together with quality of sleep perception by questionnaires evaluating income, current professional activity, highest educational degree, hypoglycaemia fear, sleepiness and potential family responsibility [15–19].

### Procedures

**Fig. 1:**
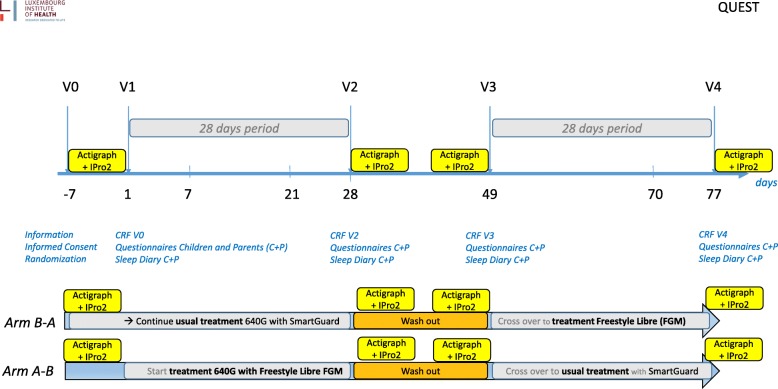
Timeline of QUEST study

**Fig. 2:**
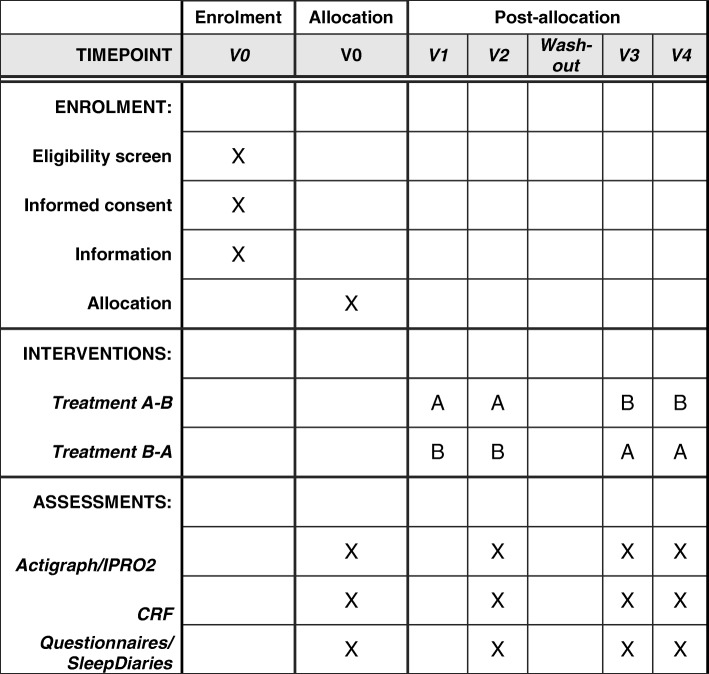
SPIRIT schedule of interventions and assessments

After they have received general information on the study aim and design, and also on the devices (pump, SmartGuard®, FreeStyle Libre® and Actigraph®), the parents and children who wish to participate in the study will be invited for the first visit (V0). The study timeline is shown in Fig. 1. The Standard Protocol Items: Recommendations for Interventional Trials (SPIRIT) schedule of interventions and assessments is shown in Fig. 2. The consent form (Additional file 1), trial questionnaires (Additional files 2, 3, 4 and 5), sleep diaries (Additional files 6, 7 and 8) and trial information (Additional files 9, 10, 11, 12, 13 and 14), as well as the populated SPIRIT checklist (Additional file 15).

At *visit V0*, after signing the informed consent/assent, the patients will be randomized to one of the two sequences (starting either with 640G with SmartGuard® or with FGM). Patients and one parent will be invited to fill out the questionnaires. A baseline HbA1c value and demographic and clinical data will be obtained in a case report form (CRF). During this period, all patients will be asked to perform a minimum of four capillary glucose measurements daily (automatically registered in their pumps). No sensor (CGM or FGM) will be used during this week. The patient and one caregiver will be provided with an Actigraph® and will be asked to fill out the sleep diaries during the 7 days of wearing the Actigraph®.

At the next visit (*V1*), the Actigraphs® will be collected for analysis. All patients will be started on the MiniMed Medtronic 640G pump, and either the SmartGuard® or the FGM will be initiated.

As all patients are pump wearers, the transition towards the Medtronic 640G pump is not complicated. The use of the two glucose measurement tools will be discussed during the dedicated training session. A 24/7 diabetes hotline will be accessible for technical or any other issues.

Settings of the SmartGuard®are standardized based on current experience [13]. The low limit will be set at 3.4 mmol/l (61 mg/dl) with an insulin suspension at ≤7.3 mmol/l (131 mg/dl) if the predicted value within 30 min is 4.5 mmol/l (81 mg/dl). An alert before low will be set on to inform the parent/patient that insulin administration is suspended. It may take time for parents to develop confidence in the new technology; therefore, in this study, we decided to include the alert before low.

At the next visit, *V2* (after 4 weeks of treatment), the patients and families will be invited to complete the questionnaires. HbA1c values will be measured, and the CRF will be completed. The IPro2® for blinded CGM will be placed for 7 days, and the patient will be instructed to perform two glucose measurements/day for calibration. The Actigraphs® will be provided. Patients and parents will be asked to fill out the sleep diaries during the following week.

This week will be followed by a washout period of 3 weeks.

During this period, the 640G pump will be maintained, but in combination with a minimum of four blood glucose measurements and no CGM or FGM.

Sleep assessment will be conducted with Actigraphs® and sleep diaries 1 week before the start of the second treatment arm.

At *visit V3*, the second treatment period will be started on either FGM or SmartGuard®.

At *visit V4*, after 4 weeks of the treatment arm, the CRF and the questionnaires will be completed, the IPro2® for blinded CGM will be placed, and again the patient will be asked to perform two blood glucose measurements per day. Actigraphs® are provided, and patients and parents will be asked to fill out the sleep diaries.

After this week the devices will be collected for analysis, and the patient will restart his/her pre-study treatment.

Ennov Clinical software will be used for data management throughout the study. For the sleep analysis, Actilife® software will be used.

### Data management and data quality

Time in glucose target will be evaluated by the blinded CGM at the end of both treatment arms.

Data will be extracted from the blinded CGM with the Medtronic GlyVaRT software tool. The pump is uploaded to transfer information to Medtronic CareLink therapy management software through the use of a Contour Next Link® glucose meter, which is also the uploading device.

Data quality will be ensured in the data management process. Double data entry will be performed in specific forms within Ennov Clinical, including online logical controls. A confrontation of both databases will be regularly carried out.

### Statistical analysis

The percent time below glucose target, < 3.0 mmol/l (54 mg/dl) and < 2.5 mmol/l (45 mg/dl), in glucose target (3.9–8 mmol/l, 70.2–144 mg/dl) and above glucose target (> 10 mmol/l, 180 mg/dl) during the final 6 days of a 5 week period will be compared between arms by using a linear model with treatment, sequence of treatments and period as fixed effects.

Sleep patterns will be assessed after sleep data validation by examining each sleep pattern. First, we will validate if the device was used a minimum of 5 days; second, we will analyse wear time and finally we will correct the sleep onset and morning wake-up according to data collected by the sleep diary.

Total sleep and wake time and number of awakenings at baseline, week 5 and week 13, in patients and at least one of their caregivers, will be analysed by using a linear mixed model with treatment given and period of treatment as fixed effects factors and patient as a random effect. The impact of family responsibility scale will be tested in the model, as well as time in target, age, gender and socio-economic status and daily physical activity.

Quality of life perception and quality of sleep (Epworth Sleepiness Scale and sleep diary) in patients and in at least one of their caregivers in the two treatment arms at baseline, week 5 and week 13 will be analysed by using a linear model or a model for categorical outcome depending on the studied outcome.

The Hypoglycaemia Index for children and the Hypoglycaemia Fear Survey for parents/caregivers at baseline, week 5 and week 13 will also be analysed with the model specific to crossover trials.

A comparison of sleep diary data versus Actigraph® data will be carried out.

Severe hypoglycaemia, defined by the International Society for Pediatric and Adolescent Diabetes (ISPAD) [12] will be analysed through a table of frequencies.

Total sleep time will be analysed using a linear mixed model with treatment given and period of treatment as fixed effects factor and patient as a random effect. Sleep analysis will be performed with Actilife® Software calculating duration of sleep during day and night and number of awakenings, in comparison with the sleep diaries.

### Sample size

Based on paediatric data, the percent time spent in glucose target (3.9–8 mmol/l) in the paediatric population is estimated to be 40–50%. Assuming that an increase of 10–15% in time in glucose target is considered as clinically meaningful, a significance level set at 5% (two sided) and a power of 80%, a minimum number of patients of 31 per group would be necessary. Taking into account the within-subject standard deviation and a maximum 10% of dropouts, a sample size of 36 patients should be included in the study.

Timeline/recruitment/checklist

Ethical approval for the final study was obtained in January 2017. Recruitment started in February 2017. The study was completed in April 2018. The timeline of the study is shown in Fig. 1. A SPIRIT checklist for this study protocol is included as Additional file 15.

## Discussion

With this study we will provide more information on whether insulin pump treatment combined with more technology (SmartGuard® feature and alerts) leads to better metabolic control. As diabetes is a chronic disease and will require lifelong treatment, the impact of quality of life and sleep may play a role in treatment adherence and outcome. The inclusion of indicators on quality of sleep with less sleep interruption, less lack of sleep and perception of quality of life in both children and their primary caregivers is essential for this study and might help to guide us to further treatment improvement.

### Trial status at time of manuscript re-submission in October 2018

Patient recruitment has been completed.

## Additional files

Additional file 1: Patient Information and consent form. (DOCX 233 kb)

Additional file 2: Questionnaires for parents. (DOC 158 kb)

Additional file 3: Questionnaires for parents. (DOC 119 kb)

Additional file 4: Questionnaires for children. (DOC 152 kb)

Additional file 5: Questionnaires for children. (DOC 146 kb)

Additional file 6: Sleep Diaries. (DOC 81 kb)

Additional file 7: Sleep Diaries. (DOC 78 kb)

Additional file 8: Sleep Diaries. (DOC 77 kb)

Additional file 9: Case Report Forms. (DOC 88 kb)

Additional file 10: Case Report Forms. (DOC 105 kb)

Additional file 11: Case Report Forms. (DOC 81 kb)

Additional file 12: Case Report Forms. (DOC 70 kb)

Additional file 13: Case Report Forms. (DOC 80 kb)

Additional file 14: Trial Protocol. (DOCX 46 kb)

Additional file 15: Spirit Checklist. (DOC 114 kb)

Additional file 16: IRB approval. (PDF 118 kb)

## Abbreviations

CGM: Continuous glucose measurement
CRF: Case report form
FGM: Flash glucose measurement
HbA1c: Haemoglobin A1c
ISPAD: International Society for Pediatric and Adolescent Diabetes
SAP: Sensor-augmented pump
SG: Sensor glucose
