# Supplementary material for: Effect of predicted low suspend pump treatment on improving glycaemic control and quality of sleep in children with type 1 diabetes and their caregivers: the QUEST randomized crossover study
Source: Trials. 2018 Dec 4;19:665. doi: 10.1186/s13063-018-3034-4 (PMC6278078; doi:10.1186/s13063-018-3034-4)
Supplement: Supplementary file 1 — Patient Information and consent form. (DOCX 233 kb) [file 13063_2018_3034_MOESM1_ESM.docx]

QUEST_______INFORMATION AND INFORMED CONSENT -

PARENTS

CLINICAL STUDY

You and your child are invited to participate in the Quest clinical study. This study will test two different continuous glucose measurement systems (Freestyle libre®, Abbott and Guardian 2 Link®, Medtronic) and their influence on the sleep and quality of life of children with type 1 diabetes and their parents.

Objective of the study

The objective of the study is to evaluate whether the Minimed 640G insulin pump (Medtronic) coupled with a subcutaneous sensor (Guardian 2 Link®, Medtronic) improves the duration and quality of sleep of your child (with type 1 diabetes) and of you as a parent. In addition we would like to analyse whether this could influence your quality of sleep. This will be compared with Minimed 640G insulin pump treatment (Medtronic) and a subcutaneous sensor not coupled to the pump (Freestyle libre®, Abbott).

Duration

The duration of the study is approximately 15 weeks. At the beginning of the study (without having changed the usual treatment), your sleep and that of your child will be evaluated by a sleep monitor (Actigraph). This device, which looks like a watch, will be worn for 7 days and 7 nights. The first 4 weeks of treatment with either system, will be followed by a 5th week of evaluation with the Actigraph (to be worn for 7 days and 7 nights by you and your child) as well as with the iPro2 (continuous glucose measurement blind control) and 3 weeks ("wash-out") with its usual treatment by insulin pump without sensor in subcutaneous. During the 3rd week of wash-out, you will wear the Actigraph again. Then your child will change treatment according to the drawing of lots before the study begins. This treatment for 4 weeks will also be followed by a week of evaluation with the Actigraph (to be worn for 7 days and 7 nights by you and your child) and with the iPro2®.

Number of medical appointments

The number of medical appointments with a member of your study team (physician or nurse) will be 5 during the study: 1 appointment during the preparatory phase, 2 appointments each at the beginning of each treatment and 2 appointments each before the treatment evaluation period on glucose control and sleep (Actigraph, iPro2® and questionnaires). The details of the visits are indicated in the Patient Course.

The conduct of the study

If your child is eligible to start the study, he or she will be randomly assigned to one of the two groups (A or B). The groups contain the same tests, only the order of processing for group A and B is different:

Visit 0 (V0)

With a team member (nurse or doctor) for the study, at the Pediatric Clinic

-

Inclusion in the study with signed consent (child and parent participating in the study)

Randomization to start with

- treatment A (Minimed 640G pump, Medtronic and sensor Enlite (Guardian 2 Link, Medtronic) or
- treatment B (Minimed 640G pump and free Freestyle® sensor, Abbott).
  - Questionnaires on the quality of sleep, quality of life and anxiety around hypoglycaemia to be completed by the child and his parent (by the one who will also wear the Actigraph).
  - Capillary glycated hemoglobin (HbA1c)
  - Explanation and start-up of the Actigraph (child and parent), to be worn for 7 days and 7 nights and returned to the study team.
  - Explanation of a "sleep diary" to be completed each morning and evening (for both child and parent) during the evaluation period with the Actigraph.

No change in treatment yet.

**Visit 1 (V1)**

Actigraphs and sleep logs return

Explanations and training for the use and implementation of the

- Treatment A (Minimed 640G Pump, Medtronic and Sensor Enlite, (Guardian 2 Link®, Medtronic) or
- Treatment B (Minimed 640G pump and Freestyle®, Abbott), at home.

Duration of this treatment: 5 weeks.

**Visit 2 (V2) 4 weeks after V1**

- Questionnaires on quality of life, quality of sleep and anxiety around hypoglycemia situations, to be completed by the child and the parent (who will also wear the Actigraph).
- Control of glycated hemoglobin (HbA1c) in capillary.
- Actigraph start-up (child and parent), to be worn for 7 days and 7 nights and then returned to the study team.
- The "sleep diary" must be completed each morning and evening (per child and per parent) during the evaluation period with the Actigraph.
- Getting the iPro2® started

⇨ After the 7-day evaluation with Actigraph and iPro2®, you will be asked to return the actigraph and I-Pro2® and treatment with the 640G insulin pump without Enlite or Freestyle sensor ("wash-out period") will be started, with a minimum of 4 blood sugar levels/day.

During the last week of these 3 weeks:

Actigraph start-up (child and parent), to be worn for 7 days and 7 nights and then returned to the study team.

**Visit 3 (V3) 4 weeks after V2**

- Capillary glycated hemoglobin (HbA1c)
- Questionnaires
- Treatment A (if treatment B at visit 1): Minimed 640G pump, Medtronic and Sensor Enlite (Guardian 2 Link®, Medtronic) or
- Treatment B (if treatment A at visit 1): Minimed 640G pump and Free Freestyle®, Abbott, at home.

Duration of this treatment: 5 weeks

**Visit 4 (V4) 4 weeks after V3**

- Questionnaires on quality of life, quality of sleep and anxiety around hypoglycaemia, to be completed by the child and parent (who will also wear the Actigraph).
- Control of glycated hemoglobin (HbA1c) in capillary.
- Actigraph start-up (child and parent), to be worn for 7 days and 7 nights and then returned to the study team.
- The "sleep diary" must be completed each morning and evening (per child and per parent) during the evaluation period with the Actigraph.
- Getting started with the iPro2®.

⇨ After the 7-day evaluation with Actigraph and iPro2, you are asked to return the actigraph and I-Pro2®, as well as the 640G. The usual treatment will be resumed.

End of the study.

Quest Study

(QUality of control and slEep in children with diabetes using new Technology)

V = visit

INFORMATION ON THE PHYSICAL ACTIVITY MONITOR

and SLEEP: INSTRUCTIONS

PLEASE REMOVE THE ACCELEROMETER ON (Day/Time) ________________________________________________.

➢ What is an accelerometer?


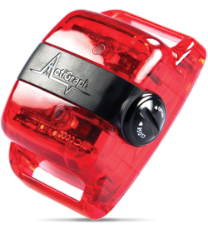
An accelerometer is a device that records information about the body's movement during daily activities such as walking. The accelerometer is safe, and uses a battery with power comparable to that of a watch. Battery life is 10 days. It can be put in contact with water for a maximum of 30 minutes at a depth of 1 meter.

This device is an instrument intended for scientific research and does not provide any data that can be directly used by the person wearing it. It is not a GPS tracking device, nor does it record heart rate. The raw data must first be downloaded using specific software before being analyzed.

➢ What am I supposed to do with the accelerometer?

1. We ask you to wear the accelerometer for 7 days, day and night (24 hours a day). It is worn on the wrist like a watch and should fit comfortably around your wrist.

2. The accelerometer is splashproof, so it can be worn with a bath or shower. However, it must be removed for swimming. If the accelerometer and wrist strap get wet, you can wipe them with a towel.

3. If you were to remove the accelerometer for any reason, remember to put it back on the same wrist as soon as possible with the black disc pointing towards your fingers.

4. Do not connect the accelerometer to an electrical or computer cable.

➢ What should I do after wearing the monitor for 7 days?

At the end of the 7-day period, we ask you to give us the accelerometer and the bracelet as well as the sensor and its reader on Sunday evening at the Pediatric Clinic or on Monday morning before 08h00. Place the accelerometer, bracelet and sensor in the envelope provided.

➢ What if I am asked about the accelerometer?

If you are asked about the accelerometer through metal detectors at airports and workplaces, you can present this fact sheet. If a security guard insists that you remove the accelerometer, simply reattach it to the same wrist after passing the security checkpoint.

➢ Who do I contact if I have a question?

If you have any questions regarding the accelerometer, please contact the DECCP team.

Evaluation during the study

Your child's study physician will perform a standard examination to measure HbA1c (capillary) levels during study visits, except at the V1 visit.

In addition it will ask you to fill in information about your child's treatment, other diseases and medications.

If your child's doctor deems it necessary, he or she will do additional tests or ask for additional tests.

Predictable risks and side effects

There are potential risks and side effects associated with the device and procedures followed. Possible additional risks are (although others are possible):

Skin irritation, swelling, discomfort, redness, bleeding, irritation, pain, rash, small "freckles" where the sensor needle was inserted, local infection at the sensor site and allergies to sensor components or dressing.

If irritation at the insertion site is observed, the sensor will be removed. It is recommended to wear the glucose sensors for 6-7 days (Guardian 2 Link® and I-Pro 2®), respectively 14 days (Freestyle Free®), depending on the protocol. If worn for a longer period it can cause such problems. To ensure correct sensor placement and to minimize insertion discomfort, an insertion device will be used to insert the sensor.

Alarms (when using the Guardian 2 Link®) can warn you that your child's glucose level is too high or too low, and by testing your child's blood glucose with a capillary sample, you can see that the value is acceptable. Nevertheless it can happen that the alarm warns you unnecessarily.

This should be discussed with your child's doctor so that alarms can be set.

Inaccurate glucose values or inappropriate alarms from the device may result in inappropriate insulin administration or carbohydrate ingestion. Such inappropriate treatment decisions may result in exacerbation of symptoms associated with hypoglycemia or hyperglycemia.

Such risks can be minimized if you and your child always follow the instructions to confirm any alarm (Guardian 2 Link®) or hypoglycemia or hyperglycemia symptoms at the Freestyle Free® by a capillary control by the glucose meter before any initiative based on the alarm or glucose values displayed.

If the risks outweigh the benefits to your child's well-being, your child's physician may decide to terminate your child's participation in the clinical study.

In order to be well informed, we recommend that you and your child ask your child's doctor any questions you may have about the risks associated with this study.

Other procedures or treatment

If you and your child do not wish to participate in this study, your child will remain with their current insulin pump therapy treatment as before. If your child does not participate, their medical care will not be affected.

Compensation and additional expenses

There will be no charge for all disposable devices and items for the duration of the study. Additional visits to your child's doctor will be made at no additional charge to you.

You and your child will not receive any financial compensation for your child's participation in this study.

Use of personal data/privacy

During participation in this study, personal information, including medical and health data, will be collected from your child's medical record. Such information may include ethnic origin, age, or biological sample identifiers collected from your child. Device information is obtained and depersonalized before data analysis (planned in collaboration with LIH, Luxemburg Institute of Health). ). Pseudonymized data will be kept for a maximum of 2 years after the end of the study.

Other designated parties involved in this study, including the third party data processor (LIH), the facility where your child is treated, the physician(s), regulatory authorities and ethics committees, may also receive and have access to your child's personal information to perform their legal and regulatory duties. The downloaded data from your child's devices will be communicated to the above mentioned parties located in the country in which your child is treated.

Your child's personal data is collected for medical research purposes, to gather information about the device and its performance during and after this study and may be used for further scientific research, for teaching and publication purposes as well as for future health studies, or to obtain current or future assessments for reimbursement of the device.

Your child's confidential personal information will be made pseudonymous and encrypted, unless this is not possible, for example if your child's name cannot be removed from a data medium, such as an electronic data capture system. In this case, the abstract - pseudonymized - including the necessary information, will be transmitted to the LIH. In any event, your child's personal information will be treated under all circumstances in accordance with appropriate privacy standards and all applicable privacy and data protection laws.

You have the right to access the personal information collected about your child and to have any inaccuracies corrected.

Voluntary Participation

Your child's participation in this study is completely voluntary. You and your child are free to refuse to participate and free to stop participation in the study at any time without fear of penalty or loss of medical care.

In addition, you and your child will be notified of any new findings that may be made during the study that may change your or your child's willingness to continue participating.

Your child's doctor may decide at any time to terminate your child's participation in the study without your prior consent. If this happens, you will be notified and the reasons will be explained.

Questions

For any questions related to the study (e.g.: risks, side effects, injury, etc.) you can contact: Dr. Schierloh, de Beaufort, and Mr. Fichelle

……………………………………………………………………………………………………………………………………………………………

Informed Consent Form Signature Sheet

I read and understood the patient information for this study and my child's doctor answered all my questions about this study.

I have had enough time to think about my child's participation in this study and know that it is entirely voluntary.

I am aware that I may decide not to participate in the study or to discontinue participation at any time without affecting the quality of my child's care or the relationship with my child's physician.

I understand and agree that personal information about my child will be collected, used and processed (manually or by computer) by the manufacturer of a medical device used in the treatment of my child or any other designated party involved in this study (e.g., hospital, physician, regulatory authorities, ethics committees).

I authorize and instruct my child's physician(s) and the institution to disclose personal information about my child.

I understand that I have the right to access the personal information collected about my child and to have any inaccuracies corrected.

I have received a copy of the Patient Information and hereby consent to my child's voluntary participation in and compliance with this study.

You may or may not agree that your child's personal physician should be informed of your child's participation in this study. Please check one option below indicating your choice:

◻ I accept that my child's personal physician will be informed of my child's participation in this study.

◻ I do not accept that my child's personal physician be informed of my child's participation in this study.

I agree to my child participating in this study.

Patient's parent (or legal guardian):

_______________________ ___________________ ________________

Name Signature Date

2.) I agree that the data collected during this study about me and my child may be used for publications and future diabetes research, and in order to use the data for research, any data that could identify me and my child will be modified and anonymized.

Patient's parent (or legal guardian):

_______________________ ___________________ ________________

Name Signature Date

Investigator or person mandated by the investigator :

I led the discussion on informed consent.

_______________________ ___________________ _________________

Name Signature

If the patient, or the patient's legal representative cannot read :

I witnessed the discussion of informed consent. I certify that the information on the consent form and any other written information has been accurately explained and apparently understood by the patient or the patient's legal representative. Informed consent was freely given by the patient or their legal representative.

Impartial witness:

_______________________ ___________________ _________________

Name Signature Date
