# Supplementary material for: Effect of predicted low suspend pump treatment on improving glycaemic control and quality of sleep in children with type 1 diabetes and their caregivers: the QUEST randomized crossover study
Source: Trials. 2018 Dec 4;19:665. doi: 10.1186/s13063-018-3034-4 (PMC6278078; doi:10.1186/s13063-018-3034-4)
Supplement: Supplementary file 2 — Questionnaires for parents. (DOC 158 kb) [file 13063_2018_3034_MOESM2_ESM.doc]

### QUESTIONNAIRE FOR THE PARENTS

Taking care of a diabetic child or teenager can be difficult for you parents for different reasons.

We thank you for participating in this study, which evaluates the effect of two glucose-monitoring instruments on the sleep and wellbeing of you and your child.

Please fill out the following questionnaire which will take you about 15 minutes.

This form asks you about the difficulties or troubles that can occur through diabetes management.

There is no right or wrong answer. We would like to know your feelings and reflexions so that we can help you by improving diabetes care in the future.

Please answer all questions.

**Thank you very much for your help! ☺ ☺**

1. Who fills out this questionnaire?

⬜ Mother ⬜ Stepmother ⬜ Father ⬜ Stepfather ⬜ Guardian

**2.**Please indicate for the following assertions, which approach best suits, your state during the last two weeks

|  | **Always** | **Almost always** | **More than half the time** | **Less than half the time** | **Almost never** | **Never** |
| --- | --- | --- | --- | --- | --- | --- |
| I was feeling happy and in a good mood | 5 | 4 | 3 | 2 | 1 | 0 |
| I was feeling calm and relaxed | 5 | 4 | 3 | 2 | 1 | 0 |
| I was feeling active and full of energy | 5 | 4 | 3 | 2 | 1 | 0 |
| I woke up fresh and available | 5 | 4 | 3 | 2 | 1 | 0 |
| My days were full and interesting | 5 | 4 | 3 | 2 | 1 | 0 |

**3.** For all the following questions on diabetic care, answer on how you manage the situation at home.

If your child takes the responsibility and/or reminds you of his care most of the time, check “my child”.

If you and your kid share the responsibility, check “my child and I”.

If you take the responsibility and/or remind him to take care, check “me”.

|  | **Who remembers or decides what to do?** | **My child** | **My child and I** | **Me** |
| --- | --- | --- | --- | --- |
| **A** | Talk about diabetes to friends |  |  |  |
| **B** | Talk about diabetes to teachers |  |  |  |
| **C** | Remember to do insulin injections |  |  |  |
| **D** | Adapt insulin level to blood test results |  |  |  |
| **E** | Decide what to eat (meals and snacks) |  |  |  |
| **F** | Have sugar in case of hypoglycaemia |  |  |  |
| **G** | Remember when to measure glycaemia |  |  |  |
| **H** | Notice changes in health e.g. weight gain  Or infection signs? |  |  |  |
| **I** | Change injection site? |  |  |  |
| **J** | Notice early signs of hypoglycaemia |  |  |  |

We would like to ask you a few questions about yourself:

**4.** What is your **highest** educational degree **obtained**?

| ☐ |  | No diploma |
| --- | --- | --- |
| ☐ |  | CATP – Certificate of Technical and Professionnal Aptitude |
| ☐ |  | CITP - Certificate of Technical and Professional Initiation |
| ☐ |  | CCM – Certificate of Manual Capability |
| ☐ |  | Diploma for Completion of Secondary Technical Studies |
| ☐ |  | Technician diploma |
| ☐ |  | Diploma for Completion of Secondary General Studies |
| ☐ |  | Bac +2 (BTS) |
| ☐ |  | Bac +3 (Bachelors/Degree) |
| ☐ |  | Bac +4 (Masters) |
| ☐ |  | Bac +5 and more (3rd Cycle, DEA, DESS, MBA, Masters, PhD, etc.) |
| ☐ |  | Diploma from a Grande Ecole, an Engineering School |
| ☐ |  | Other, please specify : .............................................................. |

**5.** What is your **current** activity?

|  | | ☐ |  | Employed | | | --- | --- | --- | --- | | ☐ |  | In school, university or in training | | | ☐ |  | Unemployed or in search of employment | | | ☐ |  | Retired or in early retirement | | | ☐ |  | At home | | | ☐ |  | Invalid | | | ☐ |  | On long-term leave (for example: illness) | | | ☐ |  | On parental leave | | | ☐ |  | Other, please specify : ............................................................... | | |  | | |  | |  | | |  | |  | | |  | |  | | |  | |
| --- | --- | --- | --- | --- | --- | --- | --- | --- | --- | --- | --- | --- | --- | --- | --- | --- | --- | --- | --- | --- | --- | --- | --- | --- | --- | --- | --- | --- | --- | --- | --- | --- | --- | --- | --- | --- | --- | --- | --- | --- | --- | --- | --- | --- | --- | --- | --- | --- | --- | --- | --- | --- | --- |

**6.** What is your monthly (or yearly) net **house income**?

*If you live with other people, add your income to that of other people living with you and with paid work, including allowances, annuities, business profits and agricultural income.*

| ☐ | Less than 750 Euros/month (less than 9000 Euros/year) |
| --- | --- |
| ☐ | 750 à 1499 Euros/month (or 9000 to 17999 Euros/year) |
| ☐ | 1500 to 2249 Euros/month (or 18000 to 26999 Euros/year) |
| ☐ | 2250 to 2999 Euros/month (or 27000 to 35999 Euros/year) |
| ☐ | 3000 to 4999 Euros/month (or 36000 to 59999 Euros/year) |
| ☐ | 5000 to 10000 Euros/month (or 60000 to 119999 Euros/year) |
| ☐ | More than 10000 Euros/month (or more than 120000 Euros/year) |
| ☐ | I don’t know |
| ☐ | I do not wish to answer |

**7.** We would like to ask you about your sleeping habits. What are the chances that you would fall asleep or doze off in the following situations?

Please focus on whether you would fall asleep, not feel tired.

Even if you haven’t been in one of the situations recently, please try to evaluate accordingly.

For each situation, say whether there was:

**0**  no chance of dozing

1. slight chance of dozing
2. moderate chance of dozing
3. high chance of dozing

| **A** | Sit and read | 0 | 1 | 2 | 3 |
| --- | --- | --- | --- | --- | --- |
| **B** | Watch television | 0 | 1 | 2 | 3 |
| **C** | Sit passively in a public place (theatre or meeting) | 0 | 1 | 2 | 3 |
| **D** | As a passenger in a car (one hour without pause) | 0 | 1 | 2 | 3 |
| **E** | Lying down in the afternoon to rest | 0 | 1 | 2 | 3 |
| **F** | Sit and have a conversation with someone | 0 | 1 | 2 | 3 |
| **G** | Sit calmly after lunch (no alcohol) | 0 | 1 | 2 | 3 |
| **H** | In an unmoving car for a few minutes in traffic | 0 | 1 | 2 | 3 |

**8.** Do you have the impression that your child’s quality of life is:

| ⬜ Very good | ⬜ Good | ⬜ Average | ⬜ Bad |
| --- | --- | --- | --- |

**9.** This questionnaire aims to better understand how people feel and behave with low glycaemia. Please answer in an honest manner to the following questions.

Below is a list of potential actions of parents of diabetic children TO AVOID LOW GLYCAEMIA and associated issues.

Read each item carefully.

Circle one of the numbers according to the probability of YOU acting in that manner.

|  |  | **Never** | **Rarely** | **Sometimes** | **Often** | **Almost always** |
| --- | --- | --- | --- | --- | --- | --- |
| **A** | Give big snacks before bed | 0 | 1 | 2 | 3 | 4 |
| **B** | Try not to leave my child alone when his glycaemia could be low | 0 | 1 | 2 | 3 | 4 |
| **C** | Allow for my child’s glycaemia to be higher in order to be safe | 0 | 1 | 2 | 3 | 4 |
| **D** | Maintain higher glycaemia if my child is alone for a while | 0 | 1 | 2 | 3 | 4 |
| **E** | As soon as my child shows symptoms of low glycaemia, give him something to eat | 0 | 1 | 2 | 3 | 4 |
| **F** | Reduce insulin doses when glycaemia seems to be too low | 0 | 1 | 2 | 3 | 4 |
| **G** | Maintain higher glycaemia when my child is going to be away for a while | 0 | 1 | 2 | 3 | 4 |
| **H** | Force him to have rapid sugar with him | 0 | 1 | 2 | 3 | 4 |
| **I** | Try for my child not to do too much exercise when his glycaemia is low | 0 | 1 | 2 | 3 | 4 |
| **J** | Frequently check my child’s glycaemia when he plans an outing | 0 | 1 | 2 | 3 | 4 |
| **K** | Get up in the middle of the night to check my child’s blood sugar levels. 0 1 2 3 4 | 0 | 1 | 2 | 3 | 4 |

**10.** Below is a list of issues parents of diabetic child can encounter.

Read each item carefully.

Circle one of the numbers according to the probability of HOW MANY TIMES YOU WORRY IN EACH SITUATION.

|  |  | **Never** | **Rarely** | **Sometimes** | **Often** | **Almost always** |
| --- | --- | --- | --- | --- | --- | --- |
| **A** | Your child doesn’t realize his glycaemia is low | 0 | 1 | 2 | 3 | 4 |
| **B** | Your child does not have food/fruit/juice with him | 0 | 1 | 2 | 3 | 4 |
| **C** | Your child feels dizzy or faints in public | 0 | 1 | 2 | 3 | 4 |
| **D** | Your child has low glycaemia during sleep | 0 | 1 | 2 | 3 | 4 |
| **E** | Your child embarrasses himself or the family during events | 0 | 1 | 2 | 3 | 4 |
| **F** | Your child has low glycaemia when alone | 0 | 1 | 2 | 3 | 4 |
| **G** | Your child seems ditsy or clumsy due to a low blood sugar | 0 | 1 | 2 | 3 | 4 |
| **H** | Your child loses control because of low glycaemia | 0 | 1 | 2 | 3 | 4 |
| **I** | There is no one around to help your child in case of low glycaemia | 0 | 1 | 2 | 3 | 4 |
| **J** | Your child makes a mistake or has an accident in school | 0 | 1 | 2 | 3 | 4 |
| **K** | Your child gets bad grades because of low glycaemia | 0 | 1 | 2 | 3 | 4 |
| **L** | Your child gets convulsions | 0 | 1 | 2 | 3 | 4 |
| **M** | Your child develops long term issues because of low glycaemia | 0 | 1 | 2 | 3 | 4 |
| **N** | Your child feels dizzy or weak | 0 | 1 | 2 | 3 | 4 |
| **O** | Your child has low glycaemia | 0 | 1 | 2 | 3 | 4 |

Please make sure you answered all questions

**Thank you very much for your help! ☺ ☺**
