# Supplementary material for: Effect of predicted low suspend pump treatment on improving glycaemic control and quality of sleep in children with type 1 diabetes and their caregivers: the QUEST randomized crossover study
Source: Trials. 2018 Dec 4;19:665. doi: 10.1186/s13063-018-3034-4 (PMC6278078; doi:10.1186/s13063-018-3034-4)
Supplement: Supplementary file 4 — Questionnaires for children. (DOC 152 kb) [file 13063_2018_3034_MOESM4_ESM.doc]

**QUESTIONNAIRE FOR CHILDREN AND TEENAGERS**

We know it can be hard to live with diabetes and want to help you the best we can. You accepted to participate in this study in which you will use various glucose monitoring instruments.

In order to better understand how these instruments might help you, we would like to ask you a few questions.

In order to answer, check the box closest to what you do, think or feel. There is no right or wrong answer so let us know what YOU think or feel. Once you have answered all the questions, place the form in the box.

**A BIG THANK YOU FOR YOUR HELP! ☺ ☺**

!


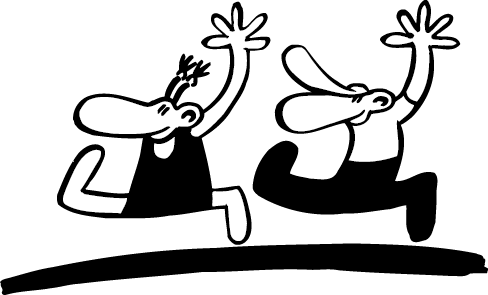


**1**. Are you with your parents for this visit? Oui  Non

**2.**

Here is a scale.

The highest level "10" amounts to the best life possible according to you,

the lowest level "0" amounts to the worst life possible

|  | 10 **best life possible** |
| --- | --- |
|  | 9 |
|  | 8 |
|  | 7 |
|  | 6 |
|  | 5 |
|  | 4 |
|  | 3 |
|  | 2 |
|  | 1 |
|  | 0 **worst life possible** |

Where would you place your current life on this scale?

Check the box next to the number that best describes your situation.

3. For all the following questions on diabetic care, answer on how you manage the situation at home.

If you take the responsibility and/or remind your parents what to do most of the time, check “me”.

If you and your parents share the responsibility, check “both, me and my parents”.

If your parents take the responsibility and/or remind you what to do most of the time, check “my parents”.

| **Who remembers and decides what to do?**  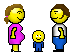 | | **Me** | **Both, me and my parents** | **My parents** |
| --- | --- | --- | --- | --- |
| **A** | Talk about diabetes to friends |  |  |  |
| **B** | Talk about diabetes to teachers |  |  |  |
| **C** | Remember to do insulin injections |  |  |  |
| **D** | Adapt insulin level to blood test results |  |  |  |
| **E** | Decide what to eat (meals and snacks) |  |  |  |
| **F** | Have sugar in case of hypoglycaemia |  |  |  |
| **G** | Remember when to measure glycaemia |  |  |  |
| **H** | Notice changes in health e.g. weight gain or infection signs? |  |  |  |
| **I** | Change injection site? |  |  |  |
| **F** | Notice early signs of hypoglycaemia |  |  |  |

**4.** The following questions refer to your usual way of life in recent times. Even if you have not done some of these things recently try to work out how they would have affected you. Use the following scale to choose the most appropriate number for each situation and circle the most appropriate number for each situation:

Chance to doze off:

**0** = I **never** doze off

**1** = I **rarely** doze off

**2** = I **often** dose doze off

**3**= I **almost always** doze off

**Question:** How likely are you to doze off or fall asleep in the following situations, **in contrast to feeling just tired?**

| **A** | Sitting and reading | 1 | 2 | 3 | 4 |
| --- | --- | --- | --- | --- | --- |
| **B** | Watching television | 1 | 2 | 3 | 4 |
| **C** | Sitting inactive in a public place (cinema, theater café) | 1 | 2 | 3 | 4 |
| **D** | As a passenger in a car for an hour without a break | 1 | 2 | 3 | 4 |
| **E** | Lying down to rest in the afternoon | 1 | 2 | 3 | 4 |
| **F** | Sitting and talking to someone | 1 | 2 | 3 | 4 |
| **G** | Sitting quietly after lunch | 1 | 2 | 3 | 4 |
| **H** | Doing homework or taking a test | 1 | 2 | 3 | 4 |

**5.** This questionnaire aims to better understand how you feel and behave with low glycaemia (hypoglycaemia). Please answer in an honest manner to the following questions, even if you haven’t lived through all the situations.

Check one of the boxes for each situation.

|  |  | **Not anxious** | **A little anxious** | **Anxious** | **Very anxious** | **Extremely anxious** |
| --- | --- | --- | --- | --- | --- | --- |
| **A** | Having low glycaemia at home with my parents | □ | □ | □ | □ | □ |
| **B** | Having low glycaemia when I sleep | □ | □ | □ | □ | □ |
| **C** | Having low glycaemia while at school | □ | □ | □ | □ | □ |
| **D** | Having low glycaemia with a group of friends | □ | □ | □ | □ | □ |
| **E** | Having low glycaemia when I’m alone | □ | □ | □ | □ | □ |
| **F** | Having low glycaemia at diabetic children’s summer camp | □ | □ | □ | □ | □ |
| **G** | Having low glycaemia at non-diabetic children summer camp | □ | □ | □ | □ | □ |
| **H** | Behaving in strangely when I have low glycaemia | □ | □ | □ | □ | □ |
| **I** | Fainting because of low glycaemia | □ | □ | □ | □ | □ |
| **J** | Being alone with no one around when I have low glycaemia | □ | □ | □ | □ | □ |
| **K** | Having a convulsion when I have low glycaemia | □ | □ | □ | □ | □ |
| **L** | Not feeling when my glycaemia is getting low | □ | □ | □ | □ | □ |
| **M** | Making mistakes at school because of low glycaemia | □ | □ | □ | □ | □ |
| **N** | Embarrassing myself because of low glycaemia | □ | □ | □ | □ | □ |
| **O** | Asking for help when my glycaemia is low | □ | □ | □ | □ | □ |
|  |  | **Never** | **Rarely** | **Half the time** | **Most of the time** | **Always** |
| **P** | I worry about having low glycaemia when my levels have been OK | □ | □ | □ | □ | □ |
| **Q** | I maintain high glycaemia levels when I’m with friends so as not to have issues because of low glycaemia | □ | □ | □ | □ | □ |
| **R** | I don’t like being alone because I worry my glycaemia might get low | □ | □ | □ | □ | □ |
| **S** | I take less insulin than necessary because I don’t want my glycaemia to get low | □ | □ | □ | □ | □ |
| **T** | I maintain higher glycaemia levels not to encounter any issues due to low glycaemia | □ | □ | □ | □ | □ |
| **U** | I check my glycaemia levels more often because I’m worried they might get low | □ | □ | □ | □ | □ |
| **V** | I want to see my doctor more often because I worry about low glycaemia | □ | □ | □ | □ | □ |
| **W** | I eat more than I need to because I don’t want to get low glycaemia | □ | □ | □ | □ | □ |
| **X** | I eat a bigger snack before bed to make sure that I won’t have low glycaemia during the night | □ | □ | □ | □ | □ |

**6.** I feel well and healthy:

always □ most of the time □ half the time □ sometimes □ rarely □

**THANK YOU VERY MUCH FOR YOUR CONTRIBUTION!! ☺ ☺**
