# Supplementary material for: Effect of predicted low suspend pump treatment on improving glycaemic control and quality of sleep in children with type 1 diabetes and their caregivers: the QUEST randomized crossover study
Source: Trials. 2018 Dec 4;19:665. doi: 10.1186/s13063-018-3034-4 (PMC6278078; doi:10.1186/s13063-018-3034-4)
Supplement: Supplementary file 6 — Sleep Diaries. (DOC 81 kb) [file 13063_2018_3034_MOESM6_ESM.doc]

**Sleep Diary**

Sufficient sleep is important for your health, well-being and happiness. When you sleep better, you feel better.

Caring for a child with diabetes can be difficult in different ways for parents. You’ve accepted to participate in the study where your child will be using different glucose monitoring systems. We would like to understand better whether these devices influence your sleep! Please, fill out this diary in the morning and in the evening during the next

week, while wearing the actigraph!

**Thank you very much!**

| **Complete in the MORNING** | | | | | | | | | | |
| --- | --- | --- | --- | --- | --- | --- | --- | --- | --- | --- |
| **Day of the week:** |  |  |  |  | |  | |  | |  |
| **Start date ----/----/----**  **Date:** | Day 1  ------- | Day 2  ------- | Day 3  ------- | Day 4  ------- | | Day 5  ------- | | Day 6  ------- | | Day 7  ------- |
| **I went to bed last night at** | PM/AM | PM/AM | PM/AM | PM/AM | | PM/AM | | PM/AM | | PM/AM |
| **I got out of bed this morning at :** | AM/PM | AM/PM | AM/PM | AM/PM | | AM/PM | | AM/PM | | AM/PM |
| **Last night I feel asleep** | | | | | | | | | | |
| Easily | □ | □ | □ | □ | □ | | □ | | □ | |
| After some time | □ | □ | □ | □ | □ | | □ | | □ | |
| With difficulty | □ | □ | □ | □ | □ | | □ | | □ | |
| **I woke up during the night:** | | | | | | | | | | |
| # of times |  |  |  |  |  | |  | |  | |
| **Last night I slept a total of :** | Hours | Hours | Hours | Hours | Hours | | Hours | | Hours | |
| **My sleep was disturbed by :**  List mental or physical factors including noise, lights, pets, allergies, temperature, discomfort, stress, etc. | | | | | | | | | | |
|  |  |  |  |  |  | |  | |  | |
| **When I woke up for the day, I felt :** | | | | | | | | | | |
| Refreshed | □ | □ | □ | □ | □ | | □ | | □ | |
| Somewhat refreshed | □ | □ | □ | □ | □ | | □ | | □ | |
| Fatigued | □ | □ | □ | □ | □ | | □ | | □ | |
| **Notes:**  Record any other factors that may affect your sleep (i.e. hours of work shift, or monthly cycle for women). |  |  |  |  |  | |  | |  | |

| **Complete at the END of the Day** | | | | | | | | | | | | | | |
| --- | --- | --- | --- | --- | --- | --- | --- | --- | --- | --- | --- | --- | --- | --- |
| **Day of the week:** | |  |  | |  |  | |  | | |  | | |  |
| **Start date ----/----/----**  **Date:** | | Day 1  ------- | Day 2  ------- | | Day 3  ------- | Day 4  ------- | | Day 5  ------- | | | Day 6  ------- | | | Day 7  ------- |
| **I consumed caffeinated drinks (e.g. coffee, cola, black/green tea, energy drinks) in the:** (M)ornig, (A)fternoon, (E)vening, (N/A) | | | | | | | | | | | | | | |
| How many cups/cans? | M |  |  |  | |  | |  | |  | | |  | |
|  | A |  |  |  | |  | |  | |  | | |  | |
|  | E | ------- | -------- | ------- | | -------- | | -------- | | -------- | | | -------- | |
| **I exercised (sports) at least 20 minutes in the:** (M)ornig, (A)fternoon, (E)vening, (N/A) | | | | | | | | | | | | | | |
|  | |  |  |  | |  |  | |  | | |  | | |
| **Medications I took today :** | |  |  |  | |  |  | |  | | |  | | |
| **Took a nap?**  (circle one) | | YES  NO | YES  NO | YES  NO | | YES  NO | YES  NO | | YES  NO | | | YES  NO | | |
| If Yes, for how long ? | |  |  |  | |  |  | |  | | |  | | |
| **During the day, how likely was I to doze off while performing daily tasks :**  No chance (1), slight chance (2), Moderate chance (3), High chance (4) | | | | | | | | | | | | | | |
| 1 / 2 / 3 / 4 | |  |  |  | |  |  | |  | | |  | | |
| **Throughout the day, my mood was…** Very pleasant (1), Pleasant (2), Unpleasant (3), Very unpleasant (4) | | | | | | | | | | | | | | |
| 1 / 2 / 3 / 4 | |  |  |  | |  |  | |  | | |  | | |
| **Approximately 2-3 hours before going to bed, I consumed:** | | | | | | | | | | | | | | |
| Alcohol | | □ | □ | □ | | □ | □ | | □ | | | □ | | |
| A heavy meal | | □ | □ | □ | | □ | □ | | □ | | | □ | | |
| Caffeine | | □ | □ | □ | | □ | □ | | □ | | | □ | | |
| Not applicable | | □ | □ | □ | | □ | □ | | □ | | | □ | | |
| **In the hour before going to sleep, my bedtime routine included:**  List activities including reading a book, using electronics, taking a bath, doing relaxation exercises, etc. | | | | | | | | | | | | | | |
|  | |  |  |  | |  |  | |  | | |  | | |
