# Supplementary material for: Effect of predicted low suspend pump treatment on improving glycaemic control and quality of sleep in children with type 1 diabetes and their caregivers: the QUEST randomized crossover study
Source: Trials. 2018 Dec 4;19:665. doi: 10.1186/s13063-018-3034-4 (PMC6278078; doi:10.1186/s13063-018-3034-4)
Supplement: Supplementary file 7 — Sleep Diaries. (DOC 78 kb) [file 13063_2018_3034_MOESM7_ESM.doc]

**Children’s Sleep Diary**

**(children below 10 years of age)**

| **Complete in the MORNING** | | | | | | | | | | |
| --- | --- | --- | --- | --- | --- | --- | --- | --- | --- | --- |
| **Day of the week** |  |  |  |  | |  | |  | |  |
| **Start date ----/----/----**  **Date:** | Day 1  ------- | Day 2  ------- | Day 3  ------- | Day 4  ------- | | Day 5  ------- | | Day 6  ------- | | Day 7  ------- |
| **My child got into bed last night at:** | PM/AM | PM/AM | PM/AM | PM/AM | | PM/AM | | PM/AM | | PM/AM |
| **My child got out of bed this morning at:** | AM/PM | AM/PM | AM/PM | AM/PM | | AM/PM | | AM/PM | | AM/PM |
| **Last night my child fell asleep:** | | | | | | | | | | |
| Easily | □ | □ | □ | □ | □ | | □ | | □ | |
| After some time | □ | □ | □ | □ | □ | | □ | | □ | |
| With difficulty | □ | □ | □ | □ | □ | | □ | | □ | |
| **My child woke up during the night:** | | | | | | | | | | |
| # of times |  |  |  |  |  | |  | |  | |
| **Last night my child slept a total of:** | Hours | Hours | Hours | Hours | Hours | | Hours | | Hours | |
| **My child’s sleep was disturbed by:**  noise, lights, pets, allergies, temperature, discomfort, nightmares, stress, pain, etc. | | | | | | | | | | |
|  |  |  |  |  |  | |  | |  | |
| **When my child woke up for the day, he/she felt :** | | | | | | | | | | |
| Rested | □ | □ | □ | □ | □ | | □ | | □ | |
| Somewhat rested | □ | □ | □ | □ | □ | | □ | | □ | |
| Tired | □ | □ | □ | □ | □ | | □ | | □ | |
| **Notes:**  Record any other factors that may have affected your child’s sleep |  |  |  |  |  | |  | |  | |

| **Complete at the END of the Day** | | | | | | | | | | | | | | | | |
| --- | --- | --- | --- | --- | --- | --- | --- | --- | --- | --- | --- | --- | --- | --- | --- | --- |
| **Day of the week** | | |  |  | | |  |  | |  | | |  | | |  |
| **Start date ----/----/----**  **Date:** | | | Day 1  ------- | Day 2  ------- | | | Day 3  ------- | Day 4  ------- | | Day 5  ------- | | | Day 6  ------- | | | Day 7  ------- |
| **My child consumed caffeinated drinks (e.g. coffee, cola, black/green tea, energy drinks) in the: (M)ornig, (A)fternoon, (E)vening, or not at all (N/A)** | | | | | | | | | | | | | | | | |
| How many cups/cans? | M | |  |  | |  | |  | |  | |  | | |  | |
|  | A | |  |  | |  | |  | |  | |  | | |  | |
|  | E | |  |  | |  | |  | |  | |  | | |  | |
| **My child exercised(sports) at least 20 minutes in the:(M)ornig, (A)fternoon, (E)vening, not at all (N/A)** | | | | | | | | | | | | | | | | |
| M / A / E / NA | | |  |  | |  | |  |  | |  | | |  | | |
| **My child took these medications** | | |  |  | |  | |  |  | |  | | |  | | |
| **Took a nap?**  (circle one) | | | YES  NO | YES  NO | | YES  NO | | YES  NO | YES  NO | | YES  NO | | | YES  NO | | |
| If Yes, for how long? | | |  |  | |  | |  |  | |  | | |  | | |
| **During the day, how likely was your child to nod off or even fall asleep while performing daily tasks :**  No chance (1), slight chance (2), Moderate chance (3), High chance (4) | | | | | | | | | | | | | | | | |
| 1/ 2/ 3/ 4 | | |  |  | |  | |  |  | |  | | |  | | |
| **Throughout the day, my child’s mood was…** Very pleasant (1), Pleasant (2), Unpleasant (3), Very unpleasant (4) | | | | | | | | | | | | | | | | |
| 1 / 2 / 3 / 4 | | |  |  | |  | |  |  | |  | | |  | | |
| **In the hour before going to sleep, my child’s bedtime routine included:**  List activities including reading a book, using electronics, taking a bath, doing relaxation exercices, etc. | | | | | | | | | | | | | | | | |
|  | |  | |  |  | | |  | |  | | |  | | |  |
