# Supplementary material for: Effect of predicted low suspend pump treatment on improving glycaemic control and quality of sleep in children with type 1 diabetes and their caregivers: the QUEST randomized crossover study
Source: Trials. 2018 Dec 4;19:665. doi: 10.1186/s13063-018-3034-4 (PMC6278078; doi:10.1186/s13063-018-3034-4)
Supplement: Supplementary file 9 — Case Report Forms. (DOC 88 kb) [file 13063_2018_3034_MOESM9_ESM.doc]

| QUALITY OF LIFE AND SLEEP V0 | | Bras de randomisation, début  : ⬜ A ⬜ B |
| --- | --- | --- |
|  | Parent: consentement signé ⬜ oui ⬜ non    d d m m y y y y  Patient: consentement signé ⬜ oui ⬜ non  HbA_1c_: . % | Date de la visite :  Diabète type 1 ⬜ oui ⬜ non |
|  | Date du diagnostic:    d d m m y y y y | Durée > à 6 mois ⬜ oui ⬜ non |
|  | Traitement par pompe depuis le  d d m m y y y y  ⬜ yes ⬜ no | Durée > à 6 mois ⬜ oui ⬜ non |

| **Date de naissance** | **Age** |
| --- | --- |
|  | ans |

**Previous Sensor use** Yes – No Duration weeks

**Previous Freestyle libre use** Yes No Duration weeks

**Wearer of Actigraph :** ☐ **mother** ☐ **father** ☐ **other**

**PUMP THERAPY (**only fill in if CSII was used the week prior to the visit)

| **Types of insulin** | **Basal insulin IU/24 hrs** | **Bolus insulin IU/24 hrs** | **No. of Bolus** |
| --- | --- | --- | --- |
| Rapid acting insulin analogue |  |  |  |

| **Hospitalisation during the last 6 months** | | **Yes** | | **Diabetes related** | **Yes no** | |
| --- | --- | --- | --- | --- | --- | --- |
| **No** | |  |  | |
| **Current infection (influencing actigraph) (severe bronchitis, vomiting, blocked nose )** cyes ⬜ no  **If “yes”: please specify:,**  **=>Parent: =>Patient:**   - **Medication taken since last visit (Specify: parent /child):**  ⬜ no | | | |  | | |
| **Blood glucose (BG) measurements: Number per day** *[ Average over the past week ]* | | | |  | | |
| **Number of Severe Hypoglycaemic episodes during the last 12 months**  *[ Resulting in unconsciousness/seizures within the last 3 months ]*  ***Any severe hypoglycemia ever*** | | | |  | | |
| **Number of Diabetic Ketoacidosis (DKA) episodes**  *[Resulting in hospital admission during the last 12 months]* | | | |  | | |
| **Concomitant pathology:** ⬜ Yes ⬜ No ( *If yes, tick below]* | | | | | | |
| ⬜ Celiac disease | ⬜ Hypothyroidism | ⬜ Hyperthyroidism | | ⬜ Other, *[Specify]:* | | |
| **Language difficulties** causing communication problems? | | | cYes cNo *If yes*  cWith the child cWith the parent | | | |
| **Other cases of type 1 diabetes in:** | | | ⬜ Father | ⬜ Mother | ⬜ Sibling | ⬜ Grandparent |
| **Other cases of type 2 diabetes in:** | | | ⬜ Father | ⬜ Mother | ⬜ Sibling | ⬜ Grandparent |
|  | | | | | | |
| **Prise de médicaments pouvant influencer le métabolisme du glucose (patient) et/ou le sommeil (parent/patient)** | | | ⬜ **oui**  ⬜ **non** |  | | |
| **Troubles physiques ou psychologiques pouvant pertuber l’étude:** cyes ⬜ no | | | |  | | |

STICKER PATIENT
