# Supplementary material for: Effect of predicted low suspend pump treatment on improving glycaemic control and quality of sleep in children with type 1 diabetes and their caregivers: the QUEST randomized crossover study
Source: Trials. 2018 Dec 4;19:665. doi: 10.1186/s13063-018-3034-4 (PMC6278078; doi:10.1186/s13063-018-3034-4)
Supplement: Supplementary file 10 — Case Report Forms. (DOC 105 kb) [file 13063_2018_3034_MOESM10_ESM.doc]

| QUALITY OF LIFE AND SLEEP V1 | Treatment arm : ⬜ A ⬜ B  **Code** |
| --- | --- |
| Parent: agreed to participate ⬜ yes ⬜ no    d d m m y y y y  Patient agreed to participate:⬜ yes ⬜ no  Sex ⬜ Male ⬜ Female | Date of Visit:  d d m m y y y y |
| Date of Birth:    d d m m y y y y    d d m m y y y y  d d m m y y y y | Date of Diagnosis |
| Height: cm Weight: . kg    d d m m y y y y | Date of first visit to  our center d d m m y y y y    our center: |

**Previous Sensor use** Yes – No Duration weeks

**Previous Freestyle libre use** Yes No Duration weeks

**Wearer of Actigraph :** ☐ **mother** ☐ **father** ☐ **other**

**PUMP THERAPY (only fill in if CSII was used the week prior to the visit)**

| **Types of insulin** | **Basal insulin IU/24 hrs** | **Bolus insulin IU/24 hrs** | **No. of Bolus** |
| --- | --- | --- | --- |
| Rapid acting insulin analogue |  |  |  |

| **Hospitalisation during the last 6 months** | | | | | **Yes** | | | | | | **Diabetes related** | | | | **Yes no** | |
| --- | --- | --- | --- | --- | --- | --- | --- | --- | --- | --- | --- | --- | --- | --- | --- | --- |
| **No** | | | | | |  | | | |  | |
| **Current infection (influencing actigraph) (severe bronchitis, vomiting, blocked nose )** cyes ⬜ no  **If “yes”: please specify:,**  **=>Parent: =>Patient:**   - **Medication taken since last visit (Specify: parent /child):**  ⬜ no | | | | | | | | | | | |  | | | | |
| **Blood glucose (BG) measurements: Number per day** *[ Average over the past week ]* | | | | | | | | | | | |  | | | | |
| **Number of Severe Hypoglycaemic episodes during the last 12 months**  *[ Resulting in unconsciousness/seizures within the last 3 months ]*  ***Any severe hypoglycemia ever*** | | | | | | | | | | | |  | | | | |
| **Number of Diabetic Ketoacidosis (DKA) episodes**  *[Resulting in hospital admission during the last 12 months]* | | | | | | | | | | | |  | | | | |
| **Concomitant pathology:** ⬜ Yes ⬜ No ( *If yes, tick below]* | | | | | | | | | | | | | | | | |
| ⬜ Celiac disease | | | ⬜ Hypothyroidism | | | ⬜ Hyperthyroidism | | | | ⬜ Other, *[Specify]:* | | | | | | |
| **Language difficulties** causing communication problems? | | | | | | | | cYes cNo *If yes*  cWith the child cWith the parent | | | | | | | | |
| **Other cases of type 1 diabetes in:** | | | | | | | ⬜ Father | | ⬜ Mother | | | | | ⬜ Sibling | | ⬜ Grandparent |
| **Other cases of type 2 diabetes in:** | | | | | | | ⬜ Father | | ⬜ Mother | | | | | ⬜ Sibling | | ⬜ Grandparent |
|  | | | | | | | | | | | | | | | | |
| **Comments :** |  |  | |  | | | | | | | | | Sticker with code patient | | | |

**HbA1C**
