# Supplementary material for: Effect of predicted low suspend pump treatment on improving glycaemic control and quality of sleep in children with type 1 diabetes and their caregivers: the QUEST randomized crossover study
Source: Trials. 2018 Dec 4;19:665. doi: 10.1186/s13063-018-3034-4 (PMC6278078; doi:10.1186/s13063-018-3034-4)
Supplement: Supplementary file 12 — Case Report Forms. (DOC 70 kb) [file 13063_2018_3034_MOESM12_ESM.doc]

| QUEST CRF V3 | Change to treatment arm : ⬜ A ⬜ B  **code** |
| --- | --- |
| d d m m y y y y  Sex ⬜ Male ⬜ Female | Date of Visit: |
| Date of Birth:    d d m m y y y y |  |
| Height: cm Weight: . kg | **Date of first visit to**  **your center:** |

**Previous Sensor use** Yes – No Wash out period

**Previous Freestyle libre use** Yes No Wash out period

**Comments:**

**PUMP THERAPY (only fill in if CSII was used the week prior to the visit)**

| **Types of insulin** | **Basal insulin IU/24 hrs** | **Bolus insulin IU/24 hrs** | **No. of Bolus** |
| --- | --- | --- | --- |
| Rapid acting insulin analogue |  |  |  |

| **Hospitalisation since the V2** | | | | **Yes** | | | | | **Diabetes related** | | | | **Yes No** | |
| --- | --- | --- | --- | --- | --- | --- | --- | --- | --- | --- | --- | --- | --- | --- |
| **No** | | | | |  | | | |  | |
| **Infection during the last week (influencing actigraph) (severe bronchitis, vomiting, blocked nose )** cyes ⬜ no  **If “yes”: please specify:,**  **=>Parent: =>Patient:** | | | | | | | | | |  | | | | |
| **Blood glucose (BG) measurements: Number per day** *[ Average over the past week ]* | | | | | | | | | |  | | | | |
| **Number of Severe Hypoglycaemic episodes since V2** | | | | | | | | | |  | | | | |
| **Number of Diabetic Ketoacidosis (DKA) episodes**  *(since V2)* | | | | | | | | | |  | | | | |
| **Concomitant pathology:** ⬜ Yes ⬜ No *[If yes, tick below ]* | | | | | | | | | | | | | | |
| ⬜ Celiac disease | | | ⬜ Hypothyroidism | | ⬜ Hyperthyroidism | | | ⬜ Other, *[Specify ]:* | | | | | | |
| **Other cases of type 1 diabetes in:** | | | | | | ⬜ Father | ⬜ Mother | | | | | ⬜ Sibling | | ⬜ Grandparent |
| **Other cases of type 2 diabetes in:** | | | | | | ⬜ Father | ⬜ Mother | | | | | ⬜ Sibling | | ⬜ Grandparent |
|  | | | | | | | | | | | | | | |
|  | | | | | | | | | | | HbA_1c_: . % | | | |
| **Comments :** |  |  | | | | | | | | | Sticker with code patient | | | |
