# Supplementary material for: Effect of predicted low suspend pump treatment on improving glycaemic control and quality of sleep in children with type 1 diabetes and their caregivers: the QUEST randomized crossover study
Source: Trials. 2018 Dec 4;19:665. doi: 10.1186/s13063-018-3034-4 (PMC6278078; doi:10.1186/s13063-018-3034-4)
Supplement: Supplementary file 14 — Trial Protocol. (DOCX 46 kb) [file 13063_2018_3034_MOESM14_ESM.docx]

**QUEST**

QUality of control and slEep in children with diabeteS

using new Technology

**Title**

Evaluation of two different glucose monitoring treatments and their impact on time in target, sleep and quality of life in children with type 1 diabetes and primary caregivers.

**Background**

Type 1 diabetes mellitus is one of the most common chronic diseases in childhood (1,2,3). These individuals require lifelong insulin treatment and optimal metabolic control, which is important to prevent short- and long-term complications (4).

To achieve optimal metabolic control the day-to-day management is challenging for the child and their family and can have a major negative impact on their quality of life (5,6).

Optimizing metabolic control in children with T1DM has led to intensified research in improved insulin pump technology and augmenting the pump data with glucose sensor information (Sensor Augmented Pumps, SAPs) to improve outcomes. Continuous interstitial glucose monitoring is associated with decreased HbA1c levels increased time in glucose target and reduced time spent in hypoglycemia in individuals with type 1 diabetes using CSII, however better outcomes are associated with longer and continued use of the sensor (7).

One of the goals of optimized therapy is to achieve fewer glycemic excursions and to maintain glucose values in the normoglycemic range.

Alerts are programmed and used in SAPs in order to inform patients and their caregivers about hypo-and hyperglycemic events to allow them to respond quickly to such glycemic excursions.

Alerts, however, can be perceived as disturbing. A new CGM device, the Freestyle Libre **®** (FGM), addresses this by measuring continuously the interstitial glucose levels, but the results can only be obtained when the patient/caregiver actively scans the sensor: no alerts are given when glucose values increase or decrease, nor will information be available when the sensor is not scanned at least every 8 hours. No communication exists between the glucose measurement and the insulin pump. The advantage of the FGM is access to 24h glucose profiles without disturbing alerts.

Without any alerts, however, the symptoms of high or low glucose levels may be missed , and intervention delayed.

Although the ultimate treatment objective is to close the loop , and automate insulin administration based on an algorithm which regulates insulin administration based on the glucose values, without the human intervention, these systems with only minimal human intervention are not yet available for routine care (8).

The 640G Medtronic ® SAP includes a further step towards closing the loop by combining alerts with an automated insulin suspension, when over the next 30 minutes the predicted glucose level will reach a pre set too low value (SmartGuard feature) Duration of the suspension is flexible and insulin administration is restarted, when glucose values are increasing again and above the low glucose range. Alerts are given in these situations, either by vibration or sound, but if no action is taken, but the insulin administration will be temporarily suspended for a minimum of 30 minutes.

Fear of nocturnal hypoglycaemia is pervasive amongst parents of children with T1DM, and is associated with heightened vigilance by parents to control regularly their children’s blood sugar values or to check the sensor information during the night. This leads to chronic sleep interruption and to lack of sleep as well for the parents as for their children with diabetes. Recent data shows 99% of parents of children with T1DM are performing blood glucose checks on their child during the night to ensure their safety whilst sleeping [9] . This highly prevalent chronic sleep interruption affects both adults with T1D and parents/carers of children with T1D with negative effects on their daily functioning and well-being (9). Anxiety and fear of hypoglycemia may have an impact on diabetes management and may complicate meeting glucose targets in patients with type 1 diabetes (10,11)

The current study, an open label single centre randomised cross over study aims to evaluate the impact of a sensor augmented pump (SAP) with a predictive algorithm to suspend temporarily insulin administration (640G**®** with the Smart Guard feature) versus the use of the same pump for isnulin administration with ‘only’ continuous glucose measurements (not interacting with the pump, Freestyle Libre **®** ) on the time in glucose target , in hypo- and hyperglycemia. As exploratory endpoints, we include the effect on sleep and quality of life in children with type 1 diabetes and their caregivers.

**Objective**

To evaluate whether sensor augmented insulin pump (Medtronic Minimed 640G ®) with the SmartGuard feature, increases time in glucose target , and improves sleep quality and quantity and quality of life perception in patients with T1DM and their primary caregivers , when compared with pump treatment with only continuous monitoring with the Freestyle Libre ®.

**Primary outcome**

- Percentage of time in glucose target (3.9-8mmol/l) during the final 6 days, of a 5 week treatment arm , measured by the blinded CGM IPro2, during at week 5 and 13, based on a between arm comparison.

**Secondary outcome**

- Percent time spent below glucose target (<3.0mmol/l and < 2,5 mmol/l) measured by blinded CGM (I-Pro 2) during week 5 and week 13
- Percent time spent above glucose target (> 10 mmol/l) measured by blinded CGM (I-Pro2) during week 5 and week 13
- Total sleep and wake time, number of awakenings measured by wireless actigraph during baseline and week 5 and week 13, in the patients and at least one of their caregivers.
- Quality of sleep in patients and in at least one of their caregivers in the two treatment arms (Epworth sleepiness scale) during baseline and week 5 and week 13,
- Quality of life perception in children during baseline and week 5 and week 13,
- Quality of life perception in parent/caregiver during baseline and week 5 and week 13,
- Childrens Hypoglycemia Index in children during baseline and week 5 and week 13,
- Hypoglycemia fear survey in parent/caregiver during baseline and week 5 and week 13,
- Family responsibility scale during baseline and week 5 and week 13
- Impact of family responsibility scale on total sleep and wake time, number of awakenings
- Impact of time in glucose target on total sleep and wake time, number of awakenings
- Impact of age, gender and SES (socio economic status) on total sleep and wake time, number of awakenings
- Daily physical activity as measured by the wireless actigraph during baseline and week 5 and week 13
- Impact of daily physical activity as measured by the actigraph, on sleep.
- Comparison of sleep diary versus actigraph data.
- Severe hypoglycemic events (11).

**Sample size**

Based on our previous report as well as other pediatric historical data , the percent time spent in glucose target (3,9-8 mmol/l) in  the pediatric population is estimated to be 40- 50 % (not published personal data, 11). Assuming that an increase of 10-15 % in time in glucose target is considered as clinically meaningful, a significance level set at 5 % (two sided) and a power of 80%, a minimum number of patients of 31 per group  would be necessary. Taking into account the within subject standard deviation and a maximum 10 % of drop out , a sample size of 36 patients should be included in the study. .

**Inclusion criteria**

- Diagnosis of type 1 diabetes (e.g. at least 1 positive antibody)
- Duration of diabetes: ≥ 6 months
- Insulin pump treatment for at least 6 months
- Age: 6 to 14 years
- HbA1c ≤ 11%
- Written informed consent of the primary caregiver, assent of the patients

**Exclusion criteria**

- No parental consent
- Physical or psychological disease likely to interfere with an appropriate conduct of the study
- Current drug therapy knowing to interfere with glucose metabolism
- Chronic sleep medication in the primary caregiver or the patient

**Study design:**

Open label single centre randomized cross over study .

The details of the study are summarized in the Patient Parcours .

The treatment arms will last 5 weeks and will be followed by a wash out period of 3 weeks before the second treatment for another 5 weeks.

During the last week of the treatment arms, the I-Pro2 **®**, will be placed for 6 days for the blinded CGM evaluation.

Before starting and during the last week of each treatment period, the subjects will wear a wireless actigraph during 24 hours , 7 nights and days.

At the first visit (V0), the study is explained, detailed information on the devices and their use are provided. When consent/assent is given, the,patients will be randomised in equal numbers to one of the two treatment sequences (First 640G with SmartGuard and second with Freestyle Libre (FGM) or first 640G with FGM and second with SmartGuard)

At the visit (V1), patients and one parent will be invited to fill out the validated questionnaires. A baseline Hba1c value and demographic and clinical data will be obtained in the CRF.

Patients will start on the 640G pump without FGM and sensor. As all patients are pump treated, the use of the new device will be a simple transition. During this period, all patients will perform a minimum of 4 capillary glucose measurements daily. The patient and one caregiver will be provided with a wireless actigraph and will be asked to fill out the sleep diaries during the 7 days of actigraph wearing. When rendering the actigraphs, either the SmartGuard or the FGM will be initiated.

Setting of the Smart Guard are standardised based on the current experience.(12) The low limit will be set at 3.4 mmol/l , with an insulin suspension at ≤7.3 mmol/l if the predicted value within 30 minutes is 4,5 mmol/l. An alert before low will be on, which will inform parent/patient that insulin administration is suspended. The insulin administration will resume automatically after maximally 2 hours , or when the glucose values -after minimally 30 minutes without insulin administration- are above the lower limit and increasing or after 2 hours. The insulin administration can be resumed manually , overriding this SmartGuard feature.

At the next visit (V2) (after 4 weeks of treatment) the patient and families will be invited to complete the questionnaires. A Hba1c will be taken and CRF will be completed. The I-Pro 2 for blinded CGM will be placed for 6 days , and the patient will be instructed to introduce 2 glucose measurements /day for calibration. The wireless actigraphs will be provided. Patients and parents will be asked to fill out the sleep diaries during the following week.

This week will be followed by a wash out period of 3 weeks.

During the wash out period, the 640G pump will be maintained, but in combination with a minimum of 4 blood glucose measurements and no sensor nor FGM.

Sleep assessment with be conducted with actigraphs and sleep diaries 1 week before the start of the second treatment arm.

The second treatment period will be started at (V3) on either the FGM or the SmartGuard feature.

After 4 weeks, at V4, the CRF and the questionnaires will be completed , the I-Pro 2 for blinded CGM, will be placed. Wireless actigraphs are provided and patients and parents will be asked to fill out the sleep diaries.

Detailed description of the study visits is provided in the ‘parcours patient.

**Study material:**

- Insulin pump: Minimed 640G, Medtronic
- Glucose sensors: Freestyle libre ®, Abbott and Enlite® sensor, Medtronic Glucometer for capillary measurements: Contour Next®, Ascensia
- iPro®2 Professional CGM, Medtronic
- Wireless sleep monitor ( LIH), Actigraph ®
- Questionnaire Children (13)
- Questionnaire Parents ( Parents/Caregiver) (14)
- Diaries
- Case Record Forms
- Time schedule
- Patient information and informed assent
- Parent Information and informed consent

Data management TBD ( LIH_DECCP)

Ennov Clinical will be used for the data management throughout the study. Ennov Clinical is a global DM software allowing the design of data entry mask, data entry, cleaning of the database through consistency edit checks, medical coding if needed with WHO DD and MEDDRA and quality control of the database before freezing.

The data entry mask will reproduce the CRF to avoid data entry errors at maximum.

Data analysis of the Actigraph will bedone in collaboration with LIH –

Gloria Aguayo, dr Ala’a AlKerwi.

**Statistical analysis (15)**

Time in glucose target will be evaluated by the blinded CGM at the end of both treatment arms.

Percent time :

- In Glucose target (3.9-8 mmol/l) during the final 6 days, of a 5 week treatment arm measured by blinded CGM during week 5 and week 13
- Below glucose target (< 3.0 mmol/l and < 2,5 mmol/l) during the final 6 days, of a 5 week treatment arm measured by blinded CGM during week 5 and week 13
- Above glucose target ( > 10 mmol/l) during the final 6 days, of a 5 week treatment arm measured by blinded CGM during week 5 and week 13

will be compared between arms by using a linear model with treatment, sequence of treatments, period as fixed effect.

Total sleep and wake time, number of awakenings at baseline, week 5 and week 13, in patients and at least one of their caregivers will be analysed using a linear model with treatment given and period of treatment as fixed effects factors and patient as a random effect.. Impact of family responsibility scale will be tested in the model, as well as time in target, age, gender and SES (socio economic status), daily physical activity.

Quality of life perception and Quality of sleep in patients and in at least one of their caregivers in the two treatment arms (Epworth sleepiness scale) at baseline, week 5 and week 13, will be analysed by using a linear model or a model for categorical outcome depending on the final type of the studied variable.

Hypoglycemia Index in children and Hypoglycemia fear survey in parent/caregiver

at baselines, week 5 and week 13, will also be analysed with the model specific to cross-over trials.

A comparison of sleep diary data versus Actigraph data will be carried out.

Severe hypoglycemia ( defined by ISPAD . (11) will be analyzed through a table of frequencies.

**References**

1. Lawrence JM, Imperatore G, Dabelea D et al. For the SEARCH for Diabetes in Youth Study Group: Trends in Incidence of Type 1 Diabetes Among Non-Hispanic White Youth in the U:S:, 2002-2009: *Diabetes.2014 Nov; 63(11):3938-3945*
2. Patterson CC, Gyürüs E, Rosenbauer J et al. Trends in childhood type 1 diabetes incidence in Europe during 1989-2008: evidence of non-uniformity over time in rates of increase: *Diabetologia 2012 Aug;55(8):2142-7.*
3. Donaghue KC, Wadwa RP, Dimeglio LA, Wong TY, Chiarelli F, Marcovecchio ML, Salem M, Raza J, Hofman PL, Craig ME. ISPAD Clinical Practise Consensus Guidelines 2014. Definition, epidemiology, and classification of diabetes in children and adolescents. International Society for Pediatric and Adolescent Diabetes: *Pediatr Diabetes. 2014 Sep;15 Suppl 20:257-69*
4. Diabetes Control and Complications Trial Research Group The effect of intensive treatment of diabetes on the development and progression of long-term complications in insulin-dependent diabetes mellitus: *N Engl J Med. 1993;329:977–86.*
5. Wood JR, Miller KMMaahs DM, et al. T1D Exchange Clinic Network. Most youth with type 1 diabetes in the T1 D Exchange Clinic Registry do not meet American Diabetes Association or International Society for Pediatric and Adolescent Diabetes clinical guidelines: *Diabetes Care 2013; 36: 2035-2037*
6. Whittemore R, Jaser S, Chao A et al. Psychological experience of parents of children with type 1 diabetes : a systematic mixed-studies review: *Diabetes Educ.* 2012 Jul-Aug;38(4):562-79.
7. Battelino T, Conget I, Olsen B et al and the SWITCH Study Group: The use and efficacy of continuous glucose monitoring in type 1 diabetes treated with insulin pump therapy: a randomised controlled trial. *Diabetologia 2012 Dec; 55(12): 3155-3162*
8. H. Thabit, M. Tauschmann, J.M. Allen, L. Leelarathna, S. Hartnell, M.E. Wilinska,

C.L. Acerini, S. Dellweg, C. Benesch, L. Heinemann, J.K. Mader, M. Holzer,

H. Kojzar, J. Exall, J. Yong, J. Pichierri, K.D. Barnard, C. Kollman, P. Cheng,

P.C. Hindmarsh, F.M. Campbell, S. Arnolds, T.R. Pieber, M.L. Evans, D.B. Dunger,

and R. Hovorka, for the APCam Consortium and AP@home Consortium. Home Use of an Artificial Beta Cellin Type 1 Diabetes. *N Engl J Med. 2015 26;373(22):2129- 40*

1. [Barnard K](http://www.ncbi.nlm.nih.gov/pubmed/?term=Barnard%20K%5BAuthor%5D&cauthor=true&cauthor_uid=26630914) [James J](http://www.ncbi.nlm.nih.gov/pubmed/?term=James%20J%5BAuthor%5D&cauthor=true&cauthor_uid=26630914), [Kerr D](http://www.ncbi.nlm.nih.gov/pubmed/?term=Kerr%20D%5BAuthor%5D&cauthor=true&cauthor_uid=26630914), [Adolfsson P](http://www.ncbi.nlm.nih.gov/pubmed/?term=Adolfsson%20P%5BAuthor%5D&cauthor=true&cauthor_uid=26630914), [Runion A](http://www.ncbi.nlm.nih.gov/pubmed/?term=Runion%20A%5BAuthor%5D&cauthor=true&cauthor_uid=26630914), [Serbedzija G](http://www.ncbi.nlm.nih.gov/pubmed/?term=Serbedzija%20G%5BAuthor%5D&cauthor=true&cauthor_uid=26630914): Impact of Chronic Sleep Disturbance for People Living With T1 Diabetes.*J Diabetes Sci Technol. 2015 Dec 1. pii: 1932296815619181. [Epub ahead of print]*
2. [Wild D](https://www.ncbi.nlm.nih.gov/pubmed/?term=Wild%20D%5BAuthor%5D&cauthor=true&cauthor_uid=17582726)1, [von Maltzahn R](https://www.ncbi.nlm.nih.gov/pubmed/?term=von%20Maltzahn%20R%5BAuthor%5D&cauthor=true&cauthor_uid=17582726), [Brohan E](https://www.ncbi.nlm.nih.gov/pubmed/?term=Brohan%20E%5BAuthor%5D&cauthor=true&cauthor_uid=17582726), [Christensen T](https://www.ncbi.nlm.nih.gov/pubmed/?term=Christensen%20T%5BAuthor%5D&cauthor=true&cauthor_uid=17582726), [Clauson P](https://www.ncbi.nlm.nih.gov/pubmed/?term=Clauson%20P%5BAuthor%5D&cauthor=true&cauthor_uid=17582726), [Gonder-Frederick L](https://www.ncbi.nlm.nih.gov/pubmed/?term=Gonder-Frederick%20L%5BAuthor%5D&cauthor=true&cauthor_uid=17582726) A critical review of the literature on fear of hypoglycemia in diabetes: Implications for diabetes management and patient education. *Patient Educ Couns. 2007 Sep;68(1):10-5*
3. Ly TT, Maahs DM, Rewers A, Dunger D, Oduwole A, Jones TW; International Society for Pediatric and Adolescent Diabetes. [**ISPAD** Clinical Practice Consensus **Guidelines** 2013. Assessment and management of **hypoglycemia** in children and adolescents with diabetes.](https://www.ncbi.nlm.nih.gov/pubmed/25040141) *Pediatr Diabetes. 2013 Sep;15 Suppl 20:180-92*
4. Abraham MB, Nicholas, JALy TT, Roby HC, Paramalingam N, Fairchild J, King BR, Ambler GF, Cameron F , Davis EA , Jones TJ Safety and efficacy of the predictive low glucose management system in the prevention of hypoglycaemia: protocol for randomised controlled home trial to evaluate the Suspend before low function. *BMJ Open2016;6:e011589*
5. Kamps JL, Roberts MC, Varela RE [Development of a new fear of **hypoglycemia** scale: preliminary results.](http://www.ncbi.nlm.nih.gov/pubmed/15784924) J Pediatr Psychol. 2005 Apr-May;30(3):287-91
6. Haugstvedt A, Wentzel-Larsen T, Aarflot M, Rokne B, Graue M. [Assessing fear of hypoglycemia in a population-based study among parents of children with type 1 **diabetes** - psychometric properties of the hypoglycemia fear survey - parent version.](http://www.ncbi.nlm.nih.gov/pubmed/25599725) BMC Endocr Disord. 2015 Jan 19;15:2. doi: 10.1186/1472-6823-15-2.
7. Senn, S. J. (2002). Cross-over Trials in Clinical Research. Chichester, Wiley.

Study group

DECCP/CHL: U Schierloh, MD, C de Beaufort MD, PhD, Muriel Fichelle, study coordinator

LIH Dept of Epidemiology A. AlKerwi MD PhD, G. Aguayo , MD , A Chiotti, MD

LIH Dept of Statistics Prof S Senn, M Vaillant PhD, A Celebic

University of Southampton Prof K Barnard
