# Supplementary material for: Effect of predicted low suspend pump treatment on improving glycaemic control and quality of sleep in children with type 1 diabetes and their caregivers: the QUEST randomized crossover study
Source: Trials. 2018 Dec 4;19:665. doi: 10.1186/s13063-018-3034-4 (PMC6278078; doi:10.1186/s13063-018-3034-4)
Supplement: Supplementary file 15 — Spirit Checklist. (DOC 114 kb) [file 13063_2018_3034_MOESM15_ESM.doc]

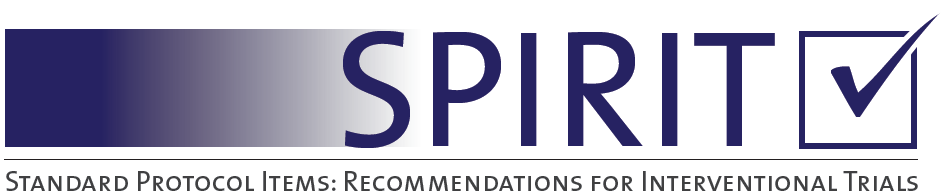


SPIRIT 2013 Checklist: Recommended items to address in a clinical trial protocol and related documents*

| Section/item | ItemNo | Description |
| --- | --- | --- |
| **Administrative information** | | |
| Title | 1 | Effect of predicted low suspend pump treatment on improving glycemic control and quality of sleep in children with type 1 diabetes and their caregiver: The QUEST randomized crossover study  Open-label, single-centre, randomised, two-period crossover study, based in the Children’s Hospital in Luxemburg |
| Trial registration | 2a +b | ClinicalTrials.gov Registration: NCT03103867  [https://clinicaltrials.gov](https://clinicaltrials.gov/) |
|  |  |  |
| Protocol version | 3 | 08 January 2017 |
| Funding | 4 | Investigator initiated study.  Medtronic contributed in kind to devices (insulin pumps and transmitters) and accessories (glucose sensors and blinded CGM) |
| Roles and responsibilities | 5a | US and CDB: concept of the study and design, protocol, recruitment , data analysis and writing of t he paper  MF and CDMD: recruitment and conduct of t he study  GA, AC and MV: study design, data management and data analysis  KB and OC: protocol |
|  | 5b | Not applicable |
|  | 5c | Medtronic contributed in kind to devices (insulin pumps and transmitters blinded and non blinded) and accessories (glucose sensors) |
|  | 5d | Not applicable |
| Introduction |  |  |
| Background and rationale | 6a | Please see description in manuscript |
|  | 6b | Please see description in manuscript |
| Objectives | 7 | Please see description in manuscript |
| Trial design | 8 | Please see text in manuscript |
| Methods: Participants, interventions, and outcomes | | |
| Study setting | 9 | Department of pediatric endocrinology and diabetology, Clinique Pédiatrique, Centre Hospitalier Luxembourg (academic hospital) |
| Eligibility criteria | 10 | Inclusion criteria   - Diagnosis of type 1 diabetes (e.g.at least 1 positive antibody) - Duration of diabetes: ≥6 months - Insulin pump treatment for at least 6 months - Age: 6 to 14 years - HbA1c≤ 11% - Written informed consent of the primary caregiver, assent of the patients   Exclusion criteria   - No parental consent - Physical or psychological disease likely to interfere with an appropriate conduct of the study - Current drug therapy knowing to interfere with glucose metabolism or continuous sleep medication |
| Interventions | 11a-d | Please see text in manuscript |
| Outcomes | 12 | Please see text in manuscript |
| Participant timeline | 13 | Timeline is attached |
| Sample size  Recruitment | 14 +15 | Based on pediatric data (16) the percent time spent in glucose target (3,9-8 mmol/l) in the pediatric population is estimated to be 40- 50 %.  Assuming that an increase of 10-15 % in time in glucose target is considered as clinically meaningful, a significance level set at 5 % (two sided) and a power of 80%, a minimum number of patients of 31 per group would be necessary. Taking into account the within subject standard deviation and a maximum 10 % of drop out, a sample size of 36 patients should be included in the study. |
| **Methods: Assignment of interventions (for controlled trials)** | | |
| Allocation: |  |  |
| Sequence generation | 16a | Method of generating the allocation sequence: computer-generated random numbers |
| Allocation concealment mechanism | 16b | Mechanism of implementing the allocation sequence: sealed envelopes |
| Implementation | 16c | Allocation sequence: Luxembourg Institute of Health (LIH), enrolment of participants and assignment of participants to interventions: Pediatric diabetologists and specialised nurses: Departement of endocrinology and diabetology, Clinique Pédiatrique, Centre Hospitalier (CHL), Luxembourg |
| Blinding (masking) | 17a | Not applicable |
|  | 17b | Not applicable |
| **Methods: Data collection, management, and analysis** | | |
| Data collection methods | 18a | Paper-based CRF’s and questionnaires (attached) |
|  | 18b | Paper-based CRF (attached) |
| Data management | 19 | Data Management in LIH (Luxembourg Institute of Health) |
| Statistical methods | 20a-c | Please see text in manuscript |
|  |  |  |
| **Methods: Monitoring** | | |
| Data monitoring/Harms | 21-b + 22 | Data monitoring and collecting reported adverse events are performed by LIH (Luxembourg Institute of Health) |
| Auditing | 23 | Not applicable |
| Ethics and dissemination | | |
| Research ethics approval | 24 | Approval obtained in January 2017, attached |
| Protocol amendments | 25 | Important protocol modifications will be submitted immediately to Êthics Committee |
| Consent or assent | 26a | Signed consent form/assent are stored in the patient files and a copy is given to the participants |
|  | 26b | Not applicable |
| Confidentiality | 27 | Personal information is stored in the secured patient data files of  the hospital. Only anonymized data are  submitted for data entry and analysis to LIH (Luxembourg Institute of Health) . Anonymized  I Pro 2 data are downloaded onto the Carelink professional data base and summarized  by Medtronic,  before submission for analysis by LIH. |
| Declaration of interests | 28 | The authors declare that they have no competing interests. |
| Access to data | 29 | Only anonymized data are submitted to LIH (Luxembourg Institute of Health) |
| Ancillary and post-trial care | 30 | Not applicable |
| Dissemination policy | 31a | Objective is to publish the results in peer reviewed journals, when possible with open access, and to communicate these to the national public. This work is a collaborate work of the CHL and LIH. French hypo fear questionnaire, after forward and backward translation and approval of the developer, will be accessible on the web (Gonder- frederick) |
|  | 31b | All those contributing to the study are included, and no professional writers are used |
|  | 31c | Plans, if any, for granting public access to the full protocol, participant-level dataset, and statistical code.: these plan have not been discussed. |
| Appendices |  |  |
| Informed consent materials | 32 | Attached |
| Biological specimens | 33 | Not applicable |

*It is strongly recommended that this checklist be read in conjunction with the SPIRIT 2013 Explanation & Elaboration for important clarification on the items. Amendments to the protocol should be tracked and dated. The SPIRIT checklist is copyrighted by the SPIRIT Group under the Creative Commons “[Attribution-NonCommercial-NoDerivs 3.0 Unported](http://www.creativecommons.org/licenses/by-nc-nd/3.0/)” license.
