# Supplementary material for: Effect of predicted low suspend pump treatment on improving glycaemic control and quality of sleep in children with type 1 diabetes and their caregivers: the QUEST randomized crossover study
Source: Trials. 2018 Dec 4;19:665. doi: 10.1186/s13063-018-3034-4 (PMC6278078; doi:10.1186/s13063-018-3034-4)
Supplement: Supplementary file 16 — IRB approval. (PDF 118 kb) [file 13063_2018_3034_MOESM16_ESM.pdf]

CNER
COMITÉ NATIONAL
D'ÉTHIQUE DE RECHERCHE
Présidente : Dr BOISANTE-BOURRIER Catherine, Médecin spécialiste en anesthésie-réanimation
Vice-président : M. Marc HASTERT, maître en droit

Membres :
Prof. ANTON Fernand, PhD, Doctorats en psychologie et en physiologie, chercheur en neurosciences comportementales
Mme BECKER-BAUER Danielle, Pharmacienne
Dr COVELLI Domenico, Médecin spécialiste en oncologie
Dr DROSTE Dirk, médecin-spécialiste en neurologie
M. ELTER Robert, chimiste
M. GLOD Frank, PhdFrank Glod, PhD Doctorat en chimie organique, expert en chimie organique et en biologie moléculaire
Dr JACOBY Chrétien, médecin spécialiste en neuro-psychatrie
Dr LE MOINE Françoise, Médecin spécialiste en oncologie
M. MAKHLOUF Abdul-Aziz, Pharmacien
Mme PETTINGER Patricia, Professeur de Philosophie
Dr SCHNEIDER Jochen, PhD, médecin spécialiste en endocrinologie et chercheur en médecine translationnelle

Dr Carine De Beaufort
CHL -Clinique Pédiatrique
4 rue Barblé
L-1210 Luxembourg

Luxembourg, 23 th April 2018

Study of title «QUEST: QUality of slEep in children with diabeteS using new Technology»

To whom it may concern,

We hereby certify that the study above mentioned has received a positive opinion (CNER N° 201609/07, effective on the 31 January 2017) of the National Research Ethics Committee (CNER) of Luxembourg.

Dr Catherine BOISANTÉ

Présidente
